# Interaction of *Haemagogus leucocelaenus* (Diptera: Culicidae) and Other Mosquito Vectors in a Forested Area, Rio de Janeiro, Brazil

**DOI:** 10.3390/tropicalmed7060094

**Published:** 2022-06-08

**Authors:** Shayenne Olsson Freitas Silva, Cecilia Ferreira de Mello, Sergio Lisboa Machado, Paulo José Leite, Jeronimo Alencar

**Affiliations:** 1 Diptera Laboratory, Oswaldo Cruz Institute (FIOCRUZ), Av. Brazil 4365, Manguinhos, Rio de Janeiro 21040-360, Brazil; shayennesilva@aluno.fiocruz.br (S.O.F.S.); ceciliamello@aluno.fiocruz.br (C.F.d.M.); paulo.leite@ioc.fiocruz.br (P.J.L.); 2 Postgraduate Program in Tropical Medicine, Oswaldo Cruz Institute (FIOCRUZ), Av. Brazil 4365, Manguinhos, Rio de Janeiro 21040-360, Brazil; 3 Postgraduate Program in Animal Biology, Institute of Biology, Federal Rural University of Rio de Janeiro, Seropédica, Rio de Janeiro 23890-000, Brazil; 4 Laboratory of Molecular Diagnosis and Hematology, Federal University of Rio de Janeiro, Rio de Janeiro 21941-901, Brazil; sergio@pharma.ufrj.br

**Keywords:** mosquito vectors, oviposition, seasonality, surveillance, yellow fever

## Abstract

The yellow fever (YF) virus has been detected throughout Brazil, with the occurrence of human cases, cyclic epizootics, and its isolation from *Haemagogus janthinomys* and *Hg. leucocelaenus*. We assessed the seasonal occurrence, egg abundancy, and oviposition interaction of mosquito vector species captured at a Private Natural Heritage Reserve in the Atlantic Forest biome. A total of 2943 eggs and 1538 mosquito larvae were collected from which 1231 belonged to entomologically important species. Ovitraps were used to collect immature mosquitoes from September 2019 to January 2021. The Mann–Whitney test was used to assess the differences in the abundance of eggs between rainy and dry seasons. Kruskal–Wallis and Dunn’s post hoc tests were used to evaluate the significance of the differences in the number of individuals from vector species. The highest percentage of mosquito vector eggs were collected during the rainy season, from December to February. Most eggs recovered from ovitraps belonged to the species *Hg. leucocelaenus*, representing 85% of all mosquito eggs identified. The other species had lower abundances and percentages: *Aedes terrens* (7%), *Haemagogus janthinomys* (5%) and *Aedes albopictus* (3%). The species that shared breeding sites with a higher frequency were *Hg. leucocelaenus* and *Hg. janthinomys*, with a statistically positive correlation (ρ = 0.74). This finding suggests that maybe the presence of *Hg. leucocelaenus* eggs acted as an attractant for *Hg. janthinomys* or vice versa. An understanding of mosquito oviposition behavior is necessary for the development of surveillance and control approaches directed against specific pathogen vectors of medical and veterinary importance.

## 1. Introduction

The maintenance of arboviruses is mainly influenced by the spatial and temporal distribution of their vectors, which in turn have their behaviors affected by different ecological settings. Hence, weather conditions, including temperature, relative humidity, and wind, impact the dispersion of females and their selection of oviposition sites [1]. For example, environmental factors, such as rainfall, can modify the ecological scenario and favor breeding conditions for mosquitoes by providing additional aquatic habitats [2].

Mosquito reproduction is affected by natural and transitory breeding sites, e.g., flooded areas, floodplains, animal dens and coconut shells, as well as by permanent or semi-permanent breeding sites including bamboo internodes and bromeliads [3]. In addition, several mosquito species are capable of breeding in water that has accumulated in tree holes, fruit peels, or even upon fallen leaves [4]. Oviposition sites are critical for the dynamics of mosquito populations and for their survival. Thus, ovitraps were used to observe alterations in the dynamics of mosquito populations, with a particular interest in oviposition behavior since it affects pathogen transmission [5]. Ovitraps were employed due to their sensitivity as a surveillance method extensively used to collect mosquito eggs in the field, even in the presence of natural breeding sites [6].

Among mosquito genera that breed in natural sites and are often associated with preserved forests are *Haemagogus* Williston, 1896, and some species of *Aedes* Meigen, 1818, such as *Ae. albopictus* Skuse, 1895 [3]. Both genera include vectors of important arboviruses, yellow fever, dengue and chikungunya, which are endemic to Brazil. These are considered by the Brazilian government as mandatory immediate notification diseases, that is, every suspected case must be promptly reported, within 24 h [7].

*Haemagogus* spp. are sylvatic mosquitoes that are found in tropical forests; they are active during the daytime and are mostly concentrated at the level of the forest canopy. In Brazil, the most hazardous sylvatic yellow fever virus (YFV) vector is *Hg. janthinomys* Dyar, 1921 [8,9]. This species has been found naturally infected with YFV in Eastern Amazonia, and in the states of Minas Gerais, Espírito Santos and Rio de Janeiro, located in the Southeast region of Brazil [10,11]. This raises concerns since epizootic YF outbreaks have apparently been shifting from the Amazon, where it is endemic, to the coast via the north–south route, through the Araguaia-Tocantins basin reaching the Central-West region of Brazil (State of Mato Grosso). Thus, YF has been reported in the northeast (State of Bahia), southeast (Minas Gerais, Espírito Santo, and São Paulo), and the southern regions of the country [12,13]. In the state of Bahia, an elevated population density of *Hg. janthinomys* was reported to be present on a road that gives access to a forest fragment near residences and is therefore dangerously close to human populations [14].

A predominance of human blood was detected in female *Hg. leucocelaenus* Dyar & Shannon, 1924, Rio Grande do Sul, Brazil. This finding demonstrates a broad range of hosts for *Hg. leucocelaenus*, as it feeds on wild animals and humans [15]. Importantly, there have already been reports of samples of this species infected with YFV in the state of Rio Grande do Sul; being considered a secondary vector of YFV in forest areas [16].

Due to their wide distribution, high abundance and natural infection, *Hg. janthinomys* and *Hg. leucocelaenus* are considered primary YFV vectors in preserved forests. We analyze the seasonal occurrence, egg abundancy, and oviposition interaction of these and other vector species to better understand the population dynamic of medically important mosquitoes.

## 2. Materials and Methods

### 2.1. Study Area

**Figure 1:**
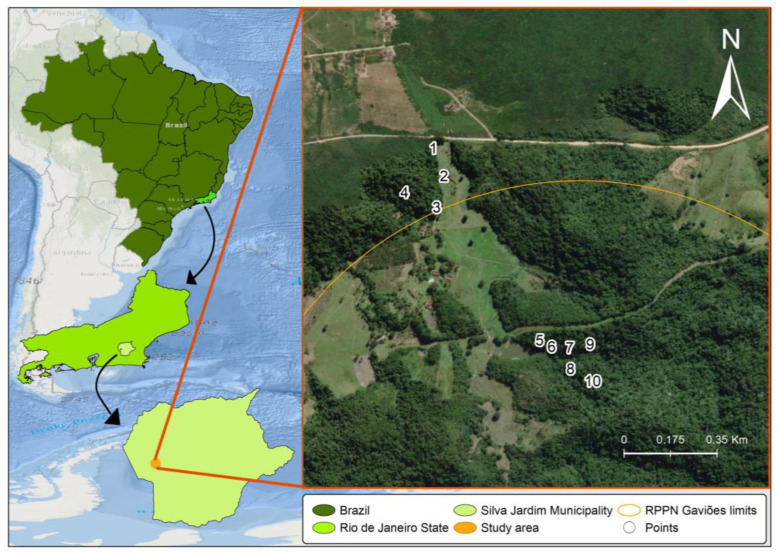
Sampling sites in the Sana Environmental Protection Area of the Private Reserve of the Gaviões Natural Heritage (RPPN Gaviões), Rio de Janeiro, Brazil. Maps were prepared using the QGIS 3.14.16 software and edited in Adobe Photoshop CS5 and CorelDraw X5. Reprinted from QGIS 3.14.16, a program under a CC BY license, with permission from Jeronimo Alencar—Fiocruz, original copyright 2021. The coordinates of the collection sites are as follows: Site 1: 22°34′08.5′′ S 42°31′39.0′′ W; Site 2: 22°34′11.9′′ S 42°31′37.7′′ W; Site 3: 22°34′15.7′′ S 42°31′38.6′′ W; Site 4: 22°34′13.9′′ S 42°31′42.5′′ W; Site 5: 22°34′31.9′′ S 42°31′25.0′′ W; Site 6: 22°34′32.7′′ S 42°31′24.6′′ W; Site 7: 22°34′32.8′′ S 42°31′22.4′′ W; Site 8: 22°34′35.4′′ S 42°31′22.3′′ W; Site 9: 22°34′32.4′′ S 42°31′19.9′′ W; Site 10: 22°34′36.9′′ S 42°31′20.1′′ W.

Samplings were carried out in an Atlantic Forest fragment of the Gaviões Private Natural Heritage Reserve (RPPN Gaviões) in Silva Jardim, Rio de Janeiro state. This private reserve comprises approximately 1600 hectares and is an Environmental Protection Area of the São João/Mico-Leão-Dourado River Basin (Figure 1). The vegetation is classified as dense ombrophilous lowland forest, one of the most threatened phytophysiognomies in the Atlantic Forest biome as a result of fragmentation [17]. Medium to advanced succession stages are predominant in this region, with characteristic secondary vegetation.

### 2.2. Ecological Analysis

Collection of mosquito immatures was performed using oviposition traps, which consisted of a black pot with a 500 mL capacity without a lid (Nutriplan Black Plastic Vase, Brazil). The study period went from September 2019 to January 2021. The data from the samplings were analyzed to determine mosquito ecological features by assessing the relationship between their populations and the seasonal distribution. The relative abundance of eggs was reported for the rainy (December, January, February) and dry (May, June, July, and August) seasons [18].

### 2.3. Collection and Rearing of Immature Culicids

A total of 10 ovitraps were installed, one trap per collection site, with three wooden oviposition paddles (2.5 cm × 14 cm) with textured surfaces, the paddles were vertically held in place by a clip. Ovitraps were filled with 300 mL of water and approximately 100 g of leaf litter. Both water and leaves were collected in bodies of water from areas near the collection sites in order to recreate a micro-ecosystem similar to natural breeding sites. Ovitraps were set at a height of 2.5 m and were fastened to the trees using nylon ropes and wire. An inspection of the traps was conducted every 20 days, and during these inspections, the water was changed and the wooden paddles replaced with new ones. The paddles were then sent to the Diptera Laboratory of the Oswaldo Cruz Institute for egg counting and subsequent immersion in transparent trays (5 cm high × 15 cm wide × 22 cm long) containing dechlorinated water. The trays were then placed in a laboratory greenhouse under a controlled experimental environment with a thermoperiod regulated at 28 °C ± 1 °C, 75–90% relative air humidity, and a 12 h day/12 h night cycle. Following a three-day period, the paddles were removed from the water and air-dried for another three days in order to quantify the hatched larvae. The immersion and air-drying processes were repeated until the hatching of all viable eggs. Larvae were fed with TetraMin flakes fish food (Tetra, Blacksburg, VA, USA), placed directly on the surface of the water, and were monitored daily. These experimental conditions enabled us to keep the specimens alive until the adult stage; species identification was carried out following the methodology described by Alencar et al. (2016) [19]. Adult mosquitoes were identified through direct observation of their morphological characteristics under a stereomicroscope (Zeiss New York, NY, USA) and using the dichotomous keys elaborated by Arnell (1973) and Forattini (2002) [4,20]. Following species identification, all specimens were submitted to the Entomological Collection of the Oswaldo Cruz Institute, FIOCRUZ.

### 2.4. Statistical Analysis

The normal distribution of the data was assessed by applying the normality test. Subsequently, the Kruskal–Wallis and Dunn’s post hoc tests were used to verify the statistical significance of differences in the numbers of individuals from medically important mosquitoes. The Mann–Whitney test was used to assess the differences in the abundance of eggs between the rainy and dry seasons. Oviposition correlation between species of the same genera, sharing the same breeding sites, was evaluated using the Spearman Correlation test. All recorded data were analyzed using software PAST version 4.09 [21].

## 3. Results

### 3.1. Seasonal Abundance of Culicid Eggs

**Figure 2:**
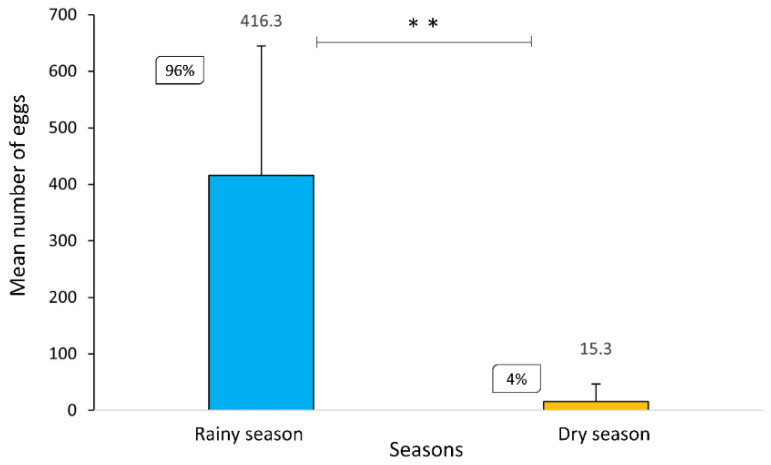
Statistically significant difference between the mean and standard deviation of the number of eggs collected in the rainy (December to March) and dry (June to August) seasons in the RPPN Gaviões (U = 0.00, Z = 2.88, *p* = 0.004). ** *p* ≤ 0.01.

A total of 2943 eggs, 1538 larvae, were collected and 1231 were identified. An emphasis was given to vector species: *Hg. leucocelaenus* (*n* = 1041), *Hg. janthinomys* (*n* = 62), *Ae. albopictus* (*n* = 40), and *Ae. terrens* (*n* = 88). The highest percentage of Culicidae eggs was registered during the rainy season, with 96%, whereas the abundance of eggs in the dry season was scarcer, with only 4%. Through the Mann–Whitney test, it was possible to observe a statistically significant difference (*p* ≤ 0.01) between the abundance of eggs in the rainy season compared to the dry season (Figure 2).

**Figure 3:**
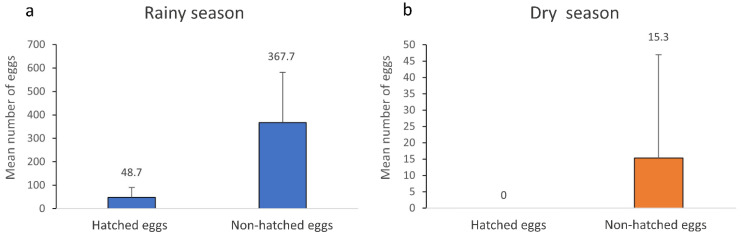
Mean and standard deviation of the number of hatched and non-hatched eggs from all Culicidae species collected from June to September 2020 in the RPPN Gaviões in the rainy (**a**) and dry (**b**) seasons.

According to the Center for Weather Forecasting and Climate Studies (CPTEC), brief heavy rains and high temperatures occur from December to February, known as the rainy season, with the dry period ranging from June to August [18]. Most of the mosquito eggs collected in both seasons were unhatched eggs (*n* = 2458). Already hatched eggs were only found during the rainy season (*n* = 292), most likely due to high temperatures and rain abundance (Figure 3a). All eggs collected in the dry season were unhatched eggs since climatic variables were probably not ideal for egg hatching. (Figure 3b).

### 3.2. Culicidae of Epidemiological Relevance

**Figure 4:**
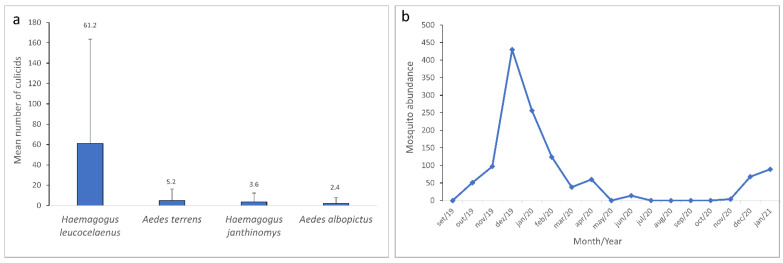
Culicidae of epidemiological concern collected from September 2019 to January 2021 in the Gaviões Reserve. (**a**) Mean and standard deviation for *Haemagogus leucocelaenus*, *Aedes terrens*, *Haemagogus janthinomys*, and *Aedes albopictus*. (**b**) Monthly mosquito abundance from September 2019 to January 2021.

Among the species of epidemiological concern, *Hg. leucocelaenus* (*n* = 1041) 85% accounted for the highest number of eggs collected, followed by *Ae. terrens* (*n* = 88) 7%, *Hg. janthinomys* (*n* = 62) 5%, and *Ae. albopictus* (*n* = 40) 3% (Figure 4a). A significant difference with a *p*-value of 0.011 (*p* ≤ 0.05) was detected among mosquito species’ abundance. These differences were observed between *Hg. leucocelaenus* and *Hg. janthinomys* (*p* = 0.009), *Hg. leucocelaenus* and *Ae. albopictus* (*p* = 0.012) and *Hg. leucocelaenus* and *Ae. terrens* (*p* = 0.003).

The highest peak of mosquito occurrence was observed in the summer month of December 2019, during the rainy season. The Culicidae population began to decline in January, still a summer month of the rainy season, with a relatively high number of individuals (*n* = 256) that continued to drop until March 2020 (*n* = 38). An increase was observed in April (*n* = 60), however, this abundance dropped considerably in May (*n* = 0), underwent a slight increment in June (*n* = 14), and fell once again (*n* = 0) for the following four months. Few culicid specimens were detected again in November 2020 (*n* = 4), and more expressive peaks were observed during the summer rainy season in December 2020 (*n* = 68) and January 2021 (*n* = 89) (Figure 4b). Through the Kruskal–Wallis test followed by Dunn’s post hoc test, it was possible to observe a statistically significant difference (*p* ≤ 0.05) in the abundance of culicids between the summer month of January and the winter month of June 2020 (*p* = 0.05).

### 3.3. Species Distribution in Oviposition Traps

**Figure 5:**
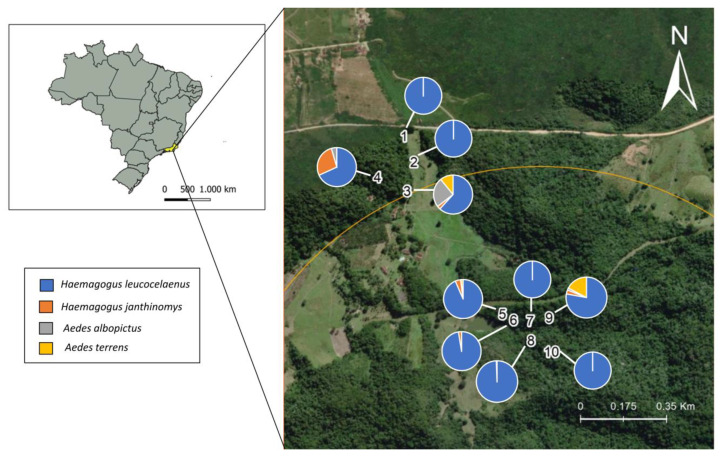
Relative frequencies of breeding sites containing immature stages of *Hg. leucocelaenus*, *Hg. janthinomys*, *Ae. albopictus* and *Ae. terrens*, collected from September 2019 to January 2021 in RPPN Gaviões.

Ovitraps with eggs were evaluated to determine the abundance of different Culicidae species. The most abundant species, found in all locations sampled, was *Hg. leucocelaenus*, being the only species found in sites 1, 2, 7 and 10 (Supplementary Table S1). *Hg. janthinomys* was found in 70% of the sites, *Aedes albopictus* in 40% and *Ae. terrens* in 30%. *Hg. leucocelaenus* and *Hg. janthinomys* were frequently found together in the same larval habitat, in 6 of the 10 collection sites. Co-occurrence of all vector species was observed for sites 3, 5, and 9 (Figure 5).

**Figure 6:**
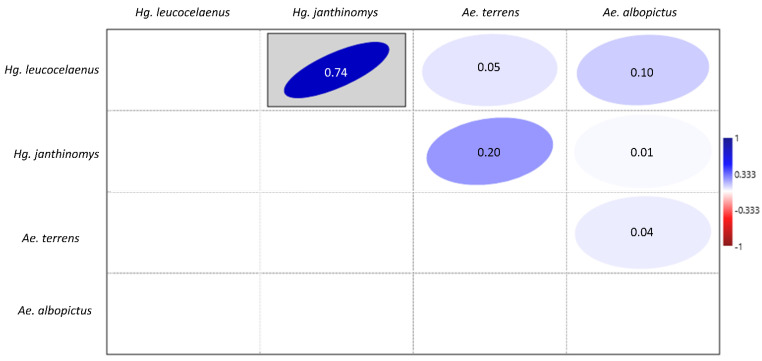
Spearman’s correlation for the abundance of eggs of Culicidae species *Hg. leucocelaenus*, *Hg. janthinomys*, *Ae. albopictus*, and *Ae. terrens* that oviposited on the same paddles from September 2019 to January 2021 in RPPN Gaviões. *p* ≤ 0.05.

Of the 30 paddles that were collected from the ovitraps, 10 contained eggs of different mosquito species. The highest number of overlapping eggs found on the same breeding site and on the same paddle were from *Hg. leucocelaenus* and *Hg. janthinomys*. These two species were found co-occurring in sites 3, 4, 5, 6, 8 and 9 (Supplementary Table S1). All of the species that performed oviposition on the same paddle were positively correlated. Hence, the Spearman’s correlation test detected a strong and statistically significant positive correlation (ρ = 0.74) between eggs from *Hg. leucocelaenus* and *Hg. janthinomys*. A positive but non-significant correlation was also observed between eggs *Ae. terrens* and *Hg. janthinomys* (ρ = 0.20), and between *Hg. leucocelaenus* and *Ae. albopictus* (ρ = 0.10) eggs (Figure 6).

## 4. Discussion

Populations of mosquitoes inhabiting fragments of the Atlantic Forest are affected by seasonality, which can therefore also impact the transmission dynamics of arboviruses [22,23].

The highest percentage of mosquito eggs were collected during the rainy season (December 2019, January, February, March, December 2020, and January 2021), with fewer mosquito eggs collected during the dry season (June, July, and August 2020). Similarly, a study conducted in the Private Natural Heritage Reserve of the Guapiaçu Ecological Reserve (REGUA) in the State of Rio de Janeiro recorded the highest numbers of mosquitoes in April and December (fall and summer, respectively), and the lowest numbers in June and October (winter and spring, respectively) [24]. However, this coincidence is not the case for other biomes, as reported by Freire et al. (2021) in a study on a fragment of seasonal dry tropical forest (Caatinga biome) of the Conservation Unit Floresta Nacional de Açu [25]. Freire et al. reported that the total number of mosquitoes collected varied widely, with September 2011 and July and May 2013 having a significantly higher abundance of culicids than in other months. This demonstrates how different biomes play an important role in the dynamics of mosquito populations across different regions of Brazil.

The most abundant species of epidemiological concern was *Hg. leucocelaenus*, representing more than 80% of all mosquito vector species identified. This species was also the most abundant during all of the seasons in the Córrego da Luz Municipal Park of Casimiro de Abreu, Rio de Janeiro state, Southeastern Brazil [26]. Additionally, the temperature was a determining factor in the increased size of *Hg. leucocelaenus* populations in a study conducted in Nova Iguaçu, Rio de Janeiro state, which showed that the likelihood of finding ovitraps containing eggs increased when the mean temperature was above 24 °C [27]. Similarly, our study detected a high frequency of *Hg. leucocelaenus* mosquitoes during the months of December 2019, January and February 2020, all of which are summer months in Brazil, characterized by high temperatures.

The diversity of mosquito oviposition behavior provides some of the most interesting examples of adaptation in the natural world. Understanding mosquito oviposition behavior is necessary for developing surveillance and control strategies against specific vectors. We observed eggs from vector mosquito species in the same breeding sites, specifically on the same paddles. Moreover, some studies suggest that the presence of congeneric or conspecific eggs can act as an attractive factor since it serves as an indicator that the breeding site is viable, has food, ideal oxygenation and temperature conditions, and an appropriate pH range for the development of immatures [28,29,30,31]. Inacio et al. (2020) reported that *Hg. spegazzinii*, shared breeding sites with *Aedes albopictus*, *Aedes terrens*, *Culex spp*., and *Toxorhynchites theobaldi* [32]. Similarly, we also observed shared breeding sites between two species of this genus, *Hg. janthinomys* and *Hg. leucocelaenus*, with the species *Ae. albopictus* and *Ae. terrens*. However, the highest overlapping oviposition was observed between the congeneric species *Hg. leucocelaenus* and *Hg. janthinomys*. This outcome may indicate that the presence of eggs from *Hg. janthinomys* acts as an attractant for *Hg. leucocelaenus* or vice versa.

## 5. Conclusions

Our results present the first record of the behavior of different YFV vector species showing overlapping oviposition on the same breeding site. This outcome demonstrates that while some species may compete when coexisting in the same larval environment, such as *Ae. aegypti* and *Culex quinquefasciatus* [32], others can have a harmonic and mutually successful interaction, as is the case between *Hg. janthinomys* and *Hg. leucocelaenus*. The summer months of the Atlantic Forest’s rainy season showed the highest peak in the number of epidemiologically important vector species such as *Hg. leucocelaenus*, a vector of the YFV. These findings demonstrate the importance of epidemiological surveillance in areas where mosquito vector species and sylvatic YFV might be circulating. Epidemiological surveillance is vital for setting off alerts for humans living or visiting the surrounding areas, specifically during the summer season in the hottest months of the year, where rainfall and mosquitoes are abundant.

## Data Availability

The dataset analyzed during the current study is available from the last author on reasonable request.

## Supplementary Materials

The following supporting information can be downloaded at: https://www.mdpi.com/article/10.3390/tropicalmed7060094/s1, Table S1: Abundance of eggs of Culicidae species Hg. leucocelaenus, Hg. janthinomys, Ae. albopictus, and Ae. terrens at the collection sites in RPPN Gaviões, from September 2019 to January 2021.
