# Supplementary material for: Interaction of Haemagogus leucocelaenus (Diptera: Culicidae) and Other Mosquito Vectors in a Forested Area, Rio de Janeiro, Brazil
Source: Trop Med Infect Dis. 2022 Jun 8;7(6):94. doi: 10.3390/tropicalmed7060094 (PMC9228385; doi:10.3390/tropicalmed7060094)
Supplement: Supplementary file 1 [file tropicalmed-07-00094-s001.zip › tropicalmed-1741771-supplementary.pdf]

Tropical Medicine and Infectious Disease

Table S1. Abundance of eggs of Culicidae species *Hg. leucocelaenus*, *Hg. janthinomys*, *Ae. albopictus*, and *Ae. terrens* at the collection sites in RPPN Gaviões, from September 2019 to January 2021.

Ovitrap	*Haemagogus leucocelaenus*	*Haemagogus janthinomys*	*Aedes albopictus*	*Aedes terrens*
1	12	0	0	0
2	17	0	0	0
3	68	3	27	12
4	75	30	5	0
5	272	13	1	5
6	76	2	0	0
7	6	0	0	0
8	183	1	0	0
9	320	13	7	71
10	12	0	0	0
**Total**	**1041**	**62**	**40**	**88**
